# Apremilast Ameliorates Experimental Arthritis *via* Suppression of Th1 and Th17 Cells and Enhancement of CD4^+^Foxp3^+^ Regulatory T Cells Differentiation

**DOI:** 10.3389/fimmu.2018.01662

**Published:** 2018-07-18

**Authors:** Weiqian Chen, Julie Wang, Zhenjian Xu, Feng Huang, Wenbin Qian, Jilin Ma, Hwa bok Wee, Gregory S. Lewis, Rayford R. June, Peter H. Schafer, Jin Lin, Song Guo Zheng

**Affiliations:** ^1^Division of Rheumatology, First Affiliated Hospital, College of Medicine, Zhejiang University, Hangzhou, China; ^2^Division of Rheumatology, Department of Medicine, Penn State University Hershey College of Medicine, Hershey, PA, United States; ^3^Department of Clinical Immunology, Third Affiliated Hospital, Sun Yat-sen Memorial Hospital of Sun Yat-sen University, Guangzhou, China; ^4^Division of Hematology, First Affiliated Hospital, College of Medicine, Zhejiang University, Hangzhou, China; ^5^Division of Rheumatology, Immunology, and Nephrology, Zhejiang Traditional Chinese Medicine and Western Medicine Hospital, Hangzhou, China; ^6^Department of Orthopaedics and Rehabilitation, Penn State University Hershey College of Medicine, Hershey, PA, United States; ^7^Translational Development, Inflammation and Immunology, Celgene Corporation, Summit, NJ, United States

**Keywords:** rheumatoid arthritis, collagen CII-induced arthritis, phosphodiesterase 4, Apremilast, regulatory T and Th17 cells

## Abstract

Apremilast is a novel phosphodiesterase 4 (PDE4) inhibitor suppressing immune and inflammatory responses. We assessed the anti-inflammatory effects of Apremilast in type II collagen (CII)-induced arthritis (CIA) mouse model. To determine whether Apremilast can ameliorate arthritis onset in this model, Apremilast was given orally at day 14 after CII immunization. Bone erosion was measured by histological and micro-computed tomographic analysis. Anti-mouse CII antibody levels were measured by enzyme-linked immunosorbent assay, and Th17, Th1 cells, and CD4^+^Foxp3^+^ regulatory T (Treg) cells were assessed by flow cytometry in the lymph nodes. Human cartilage and rheumatoid arthritis (RA) synovial fibroblasts (RASFs) implantation in the severe combined immunodeficiency mouse model of RA were used to study the role of Apremilast in the suppression of RASF-mediated cartilage destruction *in vivo*. Compared with untreated and vehicle control groups, we found that Apremilast therapy delayed arthritis onset and reduced arthritis scores in the CIA model. Total serum IgG, IgG1, IgG2a, and IgG2b were all decreased in the Apremilast treatment groups. Moreover, Apremilast markedly prevented the development of bone erosions in CIA mice by CT analysis. Furthermore, in the Apremilast treated group, the frequency of Th17 cells and Th1 cells was significantly decreased while Treg cells’ frequency was significantly increased. The high dose of Apremilast (25 mg/kg) was superior to low dose (5 mg/kg) in treating CIA. Apremilast treatment reduced the migratory ability of RASFs and their destructive effect on cartilage. Compared with the model group, Apremilast treatment significantly reduced the RASFs invasion cartilage scores in both primary implant and contralateral implant models. Our data suggest that Apremilast is effective in treating autoimmune arthritis and preventing the bone erosion in the CIA model, implicating its therapeutic potential in patients with RA.

## Introduction

Rheumatoid arthritis (RA) is a chronic inflammatory bone-destructive disorder with autoimmune features (1). It is driven by diverse cellular and humoral immune responses, resulting in articular synovial inflammation and bone destruction (2, 3). Type II collagen (CII)-induced arthritis (CIA) is a valuable animal model for investigating the pathological development of RA and for evaluating potential therapies (4). Similar to RA clinical manifestations in humans, the CIA model exhibits joint swelling, and histological examination confirms the presence of synovitis, periosteal new bone formation, articular bone erosion, and osteopenia (5). The evaluation of potential therapies requires techniques to quantify the severity of disease and pathophysiological response in experimental animals (6–8). Joint inflammation can be visually observed.

Apremilast, an oral phosphodiesterase 4 inhibitor, has been shown to regulate inflammatory mediators. Phosphodiesterase 4, the dominant phosphodiesterase expressed in immune cells, degrades cyclic AMP (cAMP) into AMP. Phosphodiesterase 4 inhibition, thereby elevates intracellular cAMP, which can downregulate the inflammatory responses through mechanisms such as partially inhibiting expression of inflammatory cytokines and increasing expression of anti-inflammatory mediators such as interleukin-10 (9).

Anti-TNF-α directed biological disease-modifying anti-rheumatic drugs currently in use (adalimumab, certolizumab pegol, etanercept, golimumab, and infliximab) are highly effective in reducing inflammation and limiting joint destruction (10). However, these treatments are insufficient and not curable (11). Side effects, such as risk for reactivation tuberculosis and malignancy, also limit their use (11, 12). Moreover, these treatments are administered *via* repeated injections with injection site reactions; hence, there is an urgent need for convenient, orally available and safe treatments that are able to reduce the production of TNF-α and other inflammatory mediators. Apremilast, an orally administered PDE4 inhibitor, has been approved in the United State and Europe since 2014 for adult patients with active psoriatic arthritis or plaque psoriasis. Apremilast mainly affects the innate immune system and also decreases the production of Th1, Th2, and Th17 cytokines, whereas its role in B cells and IL-6 production is minor. Given T effector cells (Th1 and Th17) predominately affect the pathogenesis and development of autoimmune arthritis (13, 14), we aimed to determine whether Apremilast, a PDE4 inhibitor, can prevent and even treat autoimmune arthritis through suppressing T effector cells and/or modulating regulatory T (Treg) cells.

## Materials and Methods

### Mice

Male DBA/1J mice were purchased from The Jackson Laboratory (Bar Harbor, ME, USA). C57BL/6 Foxp3^gfp^ reporter mice were generously provided by Dr. Talil Chatilla (University of California, Los Angeles). DBA/1J-FoxP3^gfp^ mice were produced by backcrossing C57BL/6 Foxp3^gfp^ mice with DBA/1J mice for 13 generations. Severe combined immunodeficiency (SCID) mice were purchased from Model Animal Research Center of Nanjing University. All animals were treated according to the National Institutes of Health guidelines for the use of experimental animal with the approval of Penn State University Hershey Medical Center and the first affiliated hospital at Zhejiang University for the Use and Care of Animals. Our experiment was followed by the standard biosecurity and institutional safety procedures in both institutes (SUZ15-01-2).

### Induction of Arthritis

Bovine CII (Condrex Inc., WA, USA) was emulsified with an equal volume of complete Freund’s adjuvant (Sigma, MO, USA) containing 4 mg/ml of heat-denatured *Mycobacterium* (BD Biosciences, CA, USA). DBA1/J and or DBA/1J-Foxp3^gfp^ mice were immunized *via* an intradermal injection at the base of the tail with 50 µl of emulsion (CII 100 μg/mouse). The mice were then euthanized by CO_2_ asphyxiation and cervical dislocation at indicated times. To determine whether Apremilast can prevent and even treat CIA, mice received Apremilast (Celgene Corporation, NJ, USA) orally daily for the continuous 10 days. Apremilast (5 or 25 mg/kg) was given to DBA/1J around 14 days after CII immunization. As a negative control, the medium alone without Apremilast (0.5% carboxymethyl cellulose, 0.25% Tween 80) was administered by oral gavage.

### Evaluation of Clinical Arthritis

Clinical signs of arthritis were evaluated every 2–3 days after immunization to determine arthritis incidence. Each paw was evaluated and scored individually for severity of arthritis using a previously described scoring system (scale 0–4) (4, 15). The scores for each paw were summed to yield a total arthritis severity score per mouse, with a maximum score of 16 for each animal. Each paw score was judged as follows: 0, no signs of arthritis, 1, mild swelling confined to the tarsal bones or ankle joint, 2, mild swelling extending from the ankle to the tarsal bones, 3, moderate swelling extending from the ankle to the metatarsal joints, and 4, severe swelling encompassing the ankle, foot, and digits, or ankylosis of the limb.

### Histopathologic Evaluation of the Joints

After the mice were sacrificed on day 56, the hind limbs were collected. Following routine fixation, decalcification, and paraffin embedding, tissue sections were prepared and stained with hematoxylin and eosin. All slides were evaluated by blinded investigators with regard to the experimental conditions. The extent of synovitis, pannus formation, and bone/cartilage destruction was determined using a graded scale, as follows (15): grade 0, no signs of inflammation; 1, mild inflammation with hyperplasia of the synovial lining without cartilage destruction; 2–4, increasing degrees of inflammatory cell infiltration and cartilage/bone destruction.

### Cell Stimulation, Suppression Assay, and Differentiation *In Vitro*

CD4^+^CD25^+^ cells (Treg) sorted from the spleen in DBA/1J mice were pretreated with 0.1 µM Apremilast or DMSO for 24 h, then cultured under a condition polarizing Th17 cells with irradiated APC (1:1), soluble anti-CD3 (1 µg/ml), soluble CD28 (1 µg/ml), and IL-6 (20 ng/ml) for another 3 days. After that, the cells were harvested, tested for IL-17a and Foxp3-GFP expression by flow cytometry. We also measured CD126 expression on Treg after pretreatment with 0.1 µM Apremilast or DMSO by flow cytometry.

To measure *in vitro* suppression, CFSE-labeled T cells were stimulated with soluble anti-CD3 (0.25 µg/ml) with irradiated APC (1:1). To the responder cells, Treg (pretreated with DMSO) or Treg (pretreated with 0.1 µM Apremilast) stimulated with or without IL-6 (20 ng/ml) was added at a 1:1 ratio, and suppression of cycling CFSE-labeled T cells was assessed flow cytometry.

### Flow Cytometry

Cells were isolated from the spleens and draining lymph nodes of arthritic mice on day 56 after CII immunization. The following fluorescence-conjugated mouse antibodies were used for flow cytometric analysis: from BioLegend (San Diego, CA, USA): mouse PerCP/Cy5.5-anti-CD4 (GK1.5), PE-CD25 (3C7), PE-IFN-γ (XMG1.2), AlexaFluor 647-IL-17a (TC11-18H10.1). Cell subset was stained with mAbs and isotype control indicated above and analyzed on a FACS Calibur flow cytometer using Cell Quest Software (Becton Dickinson). For intracellular staining, including IFN-γ and IL-17a, cells were first stained with surface marker CD4, and further fixed and permeabilized for intracellular staining. Plot figures were prepared using FlowJo Software (Tree Star Inc., Ashland, OR, USA).

### ELISA for Anti-Mouse CII Antibody

Serum levels of mouse anti-bovine CII antibody (total IgG, IgG1, IgG2a, and IgG2b, and IgG3) were measured according to a modification of the previously published protocol (16). Briefly, coated 96-well plates were coated with 1 µg/ml bovine CII and incubated at 4°C overnight, followed by blocking with a 2 h incubation at 4°C with 3% BSA in phosphate-buffered saline (PBS, pH 7.4). Serum samples diluted at 1:1,000 in PBS containing 1% BSA were incubated 1.5 h at 37°C. Goat anti-mouse IgG, IgG1, IgG2a, IgG2b, and IgG3 conjugated to HRP (Southern Biotechnology, Birmingham, AL, USA) diluted at 1:4,000 were incubated for 1.5 h at 37°C to detect anti-CII antibody isotypes and IgG subclasses. A pre-warmed *o*-phenylenediamine buffer was used to visualize the reaction. The optical density at 490 nm was read with a Molecular Devices plate reader (Molecular Devices, Menlo Park, CA, USA). Antibody levels were expressed as units with reference to a standard serum.

### Micro-Computed Tomographic Image Analysis

Mice were anesthetized with 2% isoflurane. The high-resolution micro-computed tomography (micro-CT) system (VivaCT 40, Scanco, Switzerland) was used to acquire *in vivo* imaging of the three-dimensional bone. The scans were performed with 3.6 mm length including entire single mouse foot with the following parameters: 17.5 µm voxel size at 55 kV, 145 µA, 200 ms integration time, 211 image slices. The micro-CT images were converted to 8-bit, imported into Mimics software (Materialise, Belgium), then filtered using discrete Gaussian filtering (variance = 1; max kernel width = 1). Volumes of interest in the metatarsophalangeal joint were used to quantify bone erosion as follows. Second through fourth metatarsal and phalangeal bones were segmented from surrounding soft tissue using a consistent image intensity threshold. Three volumes of interest were set with ±1 mm length in the distal and the proximal direction from the center of each metatarsophalangeal joint. These volumes of interest were oriented consistently based on the 3D longitudinal axis of the third metatarsal. The bone volumes of the three metatarsophalangeal joints were then calculated.

### Inflamed Synovial Tissue-Mediated Humanized Animal Model

Sponge-cartilage complex, containing a piece of cartilage with RA synovial fibroblasts (RASFs, 5 × 10^5^) from RA patients was implanted into the flank skin of an SCID mouse (primary implant). Surgery was performed on the condition of isoflurane anesthesia. The skin incision was made with a sterile scalpel. One drop of bupivacaine was applied to the incision before closure for local analgesia. The mice were monitored for signs of discomfort. Two RA patients (one female, 55 years old, with RA for 12 years, another female, 54 years old, with RA for 3 years) were recruited from the first affiliated hospital at Zhejiang University with IRB approval. The written informed consent was obtained from these two patients. We also inserted cartilage without RASFs under the skin of the contralateral flank (contralateral implant). After the humanized synovitis model had implanted, Apremilast (25 mg/kg, once daily) was given to SCID mice on the day when cartilage with RASFs was implanted for the continuous 10 days. We removed the implants after 60 days for evaluation. They were stained by standard hematoxylin and eosin. Invasion scores were classified as a previous report (17).

### Statistics

Data are expressed as mean ± SEM. Data were analyzed using the unpaired *t*-tests (Mann–Whitney) between two groups or one-way ANOVA for comparison among multiple groups followed by Turkey’s test as appropriate. Differences were considered statistically significant when *p* < 0.05.

## Results

### Apremilast Therapy Delayed Arthritis Onset and Reduced Arthritis Scores in CIA Model

**Figure 1:**
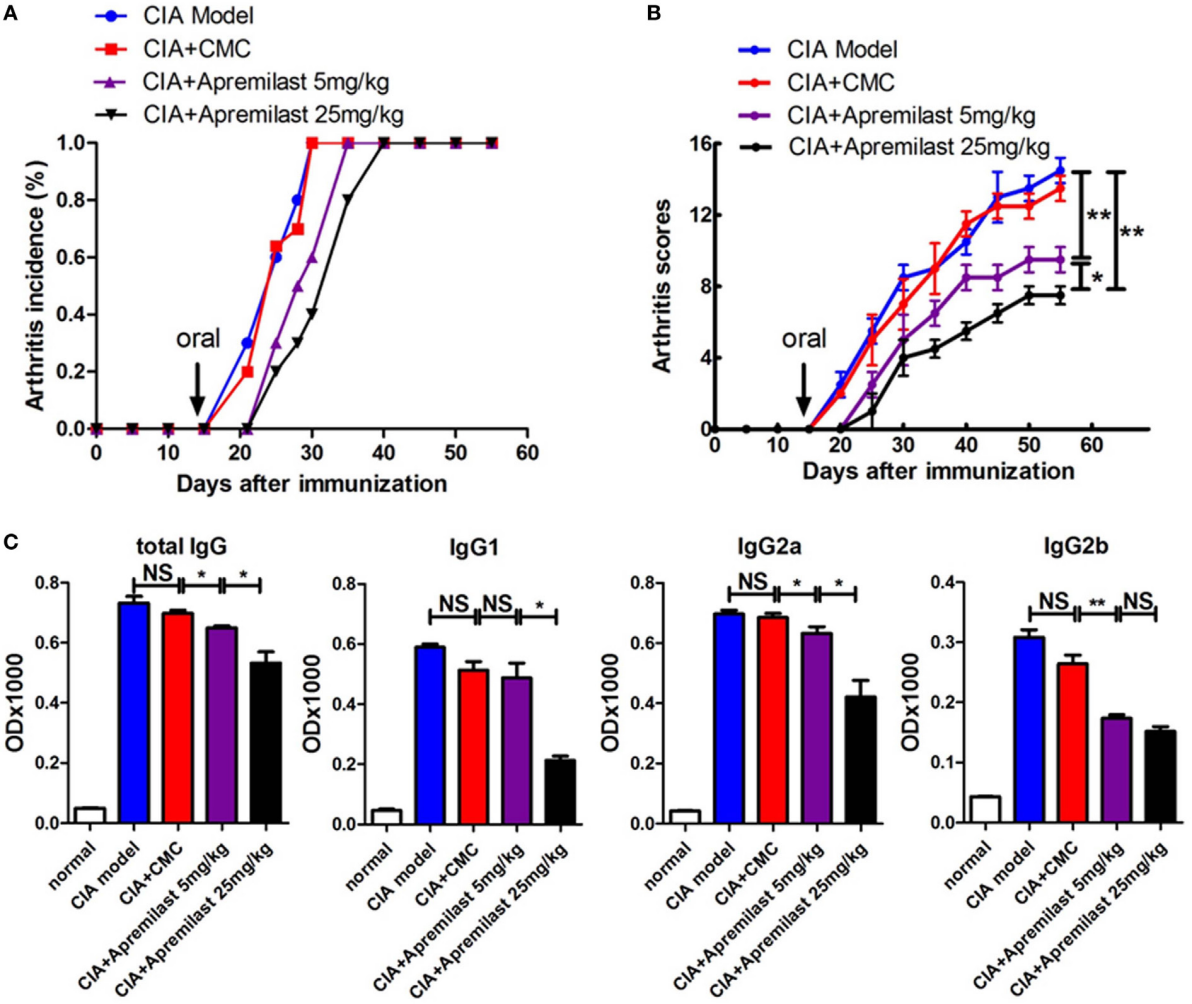
Apremilast delayed arthritis onset and reduced arthritis scores in the collagen-induced arthritis (CIA) model. DBA/1J-FoxP3^gfp^ mice were immunized with type II collagen emulsified with Freund’s complete adjuvant. On day 14 after immunization, Apremilast (5 or 25 mg/kg) was given orally once daily for 10 days. Vehicle alone (0.5% carboxymethyl cellulose, 0.25% Tween 80, CMC) was administered by oral gavage as a negative control. **(A)** The incidence of arthritis and **(B)** arthritis severity scores were determined at various time points after immunization. **(C)** Total serum IgG, IgG1, IgG2a, and IgG2b anti-mouse type II collagen antibody levels were measured by enzyme-linked immunosorbent assay. The data indicate the mean ± SEM of five mice per group from two independent experiments. Data were analyzed using the one-way ANOVA for comparison among multiple groups, followed by Turkey’s test (*p < 0.05, **p < 0.01).

Apremilast has been shown to regulate inflammatory mediators. We first tested whether Apremilast (5 or 25 mg/kg) can change arthritis onset. Both doses of Apremilast significantly delayed arthritis onset and markedly reduced arthritis scores in the CIA model. High dose of Apremilast (25 mg/kg) was superior to low dose (5 mg/kg) in reducing arthritis scores (Figures 1A,B). We then examined serum anti-CII antibody and found that the total serum IgG, IgG2a, and IgG2b were decreased in the Apremilast (5 mg/kg) treatment group, compared with the vehicle control group. In a dose response, the total serum IgG and IgG2a levels were lower in the Apremilast (25 mg/kg) group compared with the Apremilast (5 mg/kg) group. IgG1 was only decreased in the Apremilast (25 mg/kg) group (Figure 1C), but IgG3 did not change in the Apremilast treatment groups (data not shown).

### Apremilast Reduced Arthritis and Prevented Bone Erosion in CIA Model

**Figure 2:**
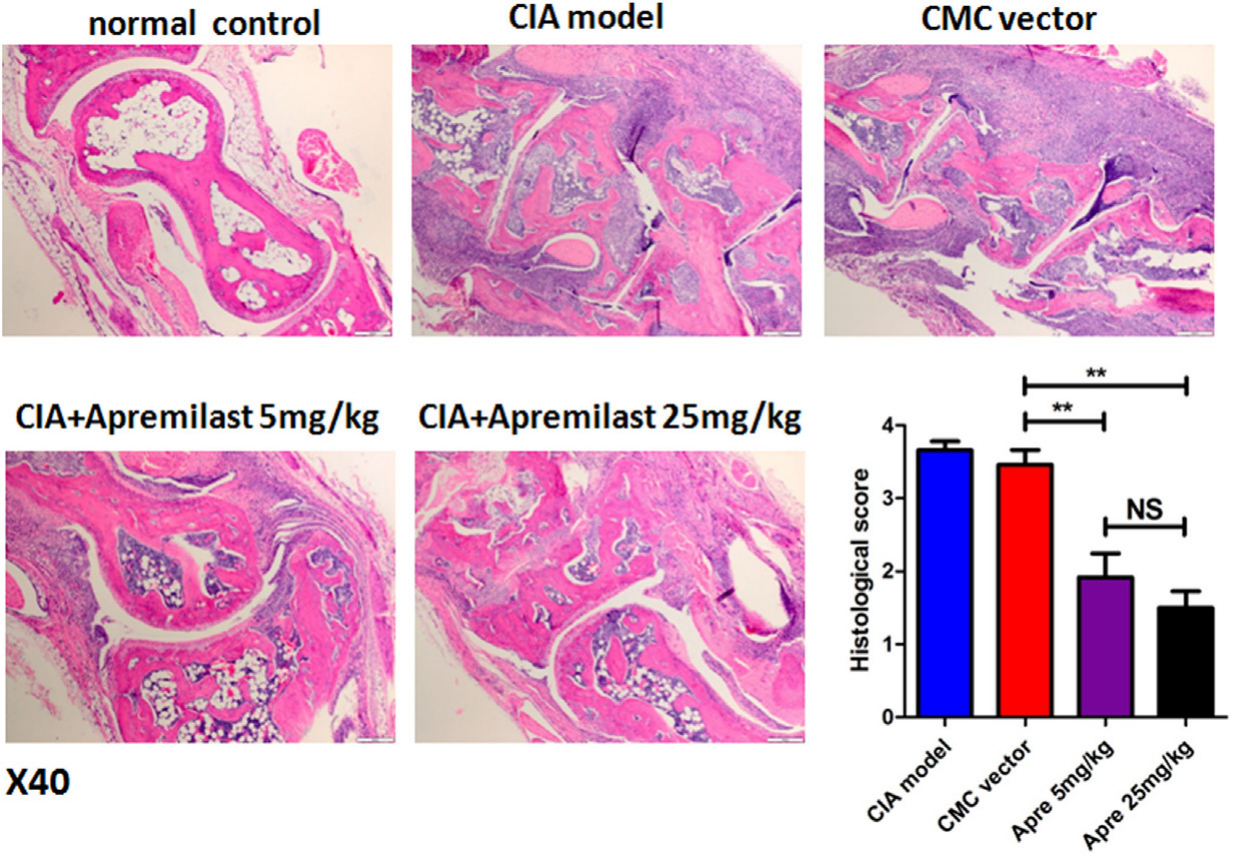
Apremilast administration reduced arthritis pathology in collagen-induced arthritis (CIA) model. Ankle joint sections were stained with hematoxylin and eosin 56 days after the primary immunization and evaluated for the histopathologic features of synovitis, pannus, and erosion (representative results were shown; 40×) in Apremilast (5 or 25 mg/kg), CMC vehicle alone, CIA model, and normal groups. Results were expressed quantitatively as the histopathology score (right lower panel). The data indicate the mean ± SEM of two separated experiments, each group with five mice. Data were analyzed using the one-way ANOVA for comparison among multiple groups, followed by Turkey’s test (**p < 0.01).

**Figure 3:**
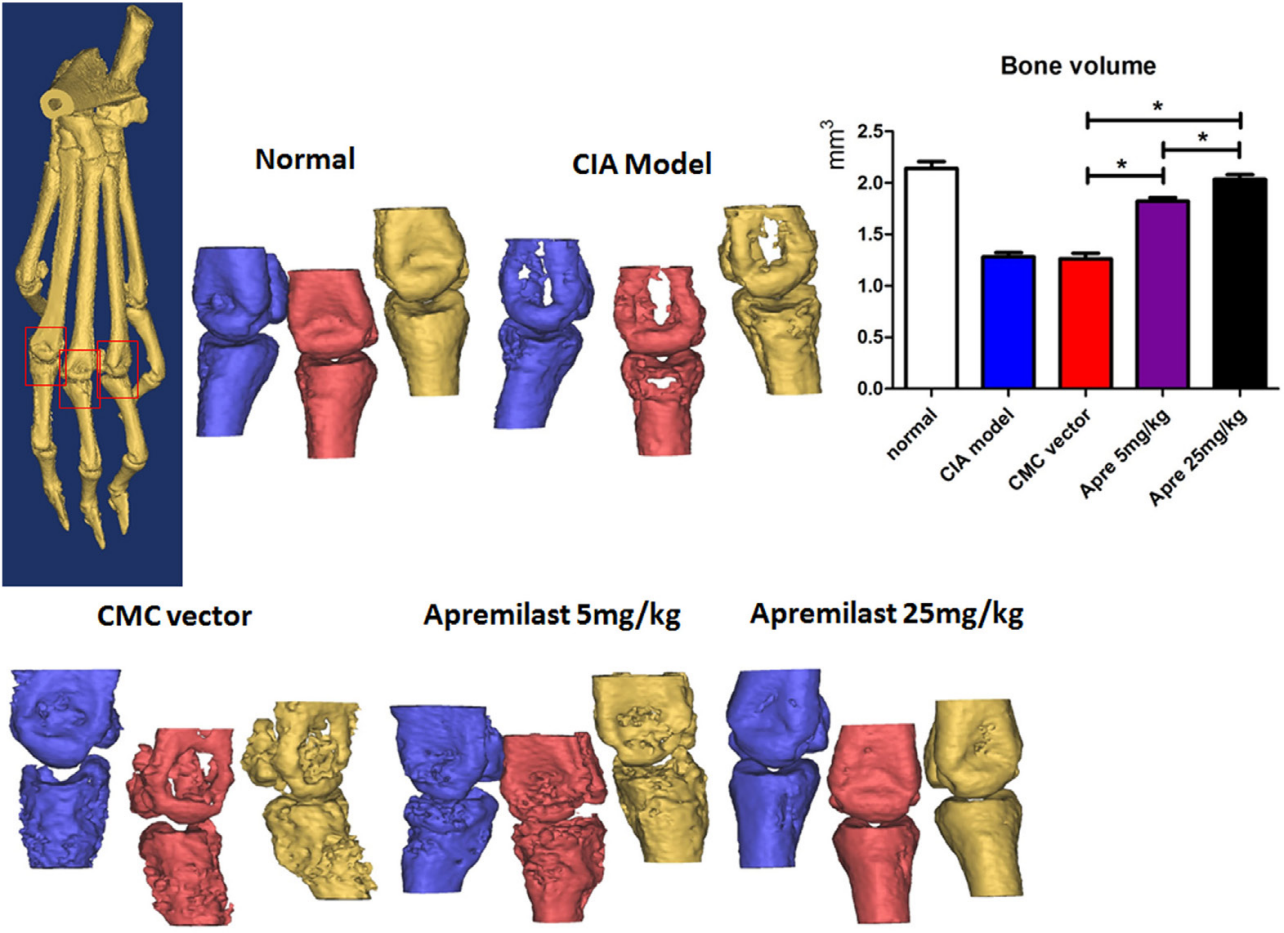
Apremilast prevented bone erosion in collagen-induced arthritis (CIA) model. Apremilast (5 or 25 mg/kg), CMC vehicle alone, CIA model, and normal groups mice were killed on day 56, the right hind limbs were collected and analyzed by the high-resolution micro-computed tomography (micro-CT) system (VivaCT 40). Second through fourth metatarsal and phalangeal bones were segmented from surrounding soft tissue using a consistent image intensity threshold. Three volumes of interest were set with ±1 mm length in the distal and proximal direction from the center of each metatarsophalangeal joint. The bone volumes of the three metatarsophalangeal joints were then calculated. Representative micro-CT images were shown. The summary data are shown in the left panel. The data indicate the mean ± SEM of five mice per group from two independent experiments (*p < 0.05). Data were analyzed using the one-way ANOVA for comparison among multiple groups, followed by Turkey’s test.

To determine whether Apremilast can prevent CIA mice from bone erosion, joint HE staining and micro-CT scan was applied. Histological changes in the whole ankle joints demonstrated a significant decrease in synovitis, pannus formation, and destruction of bone and cartilage after treatment with Apremilast (Figure 2). There was no difference between the lower dose group and the high dose group for histological findings (Figure 2). Quantitative analyses of the whole ankle confirmed a similarly significant increase in the bone volume of the metatarsophalangeal joint in the Apremilast treatment group by micro-CT analysis, compared with vehicle control (Figure 3).

### Apremilast Reduced Arthritis Through Suppression of Th1 and Th17 Cells and Enhancement of Treg Cells Differentiation

**Figure 4:**
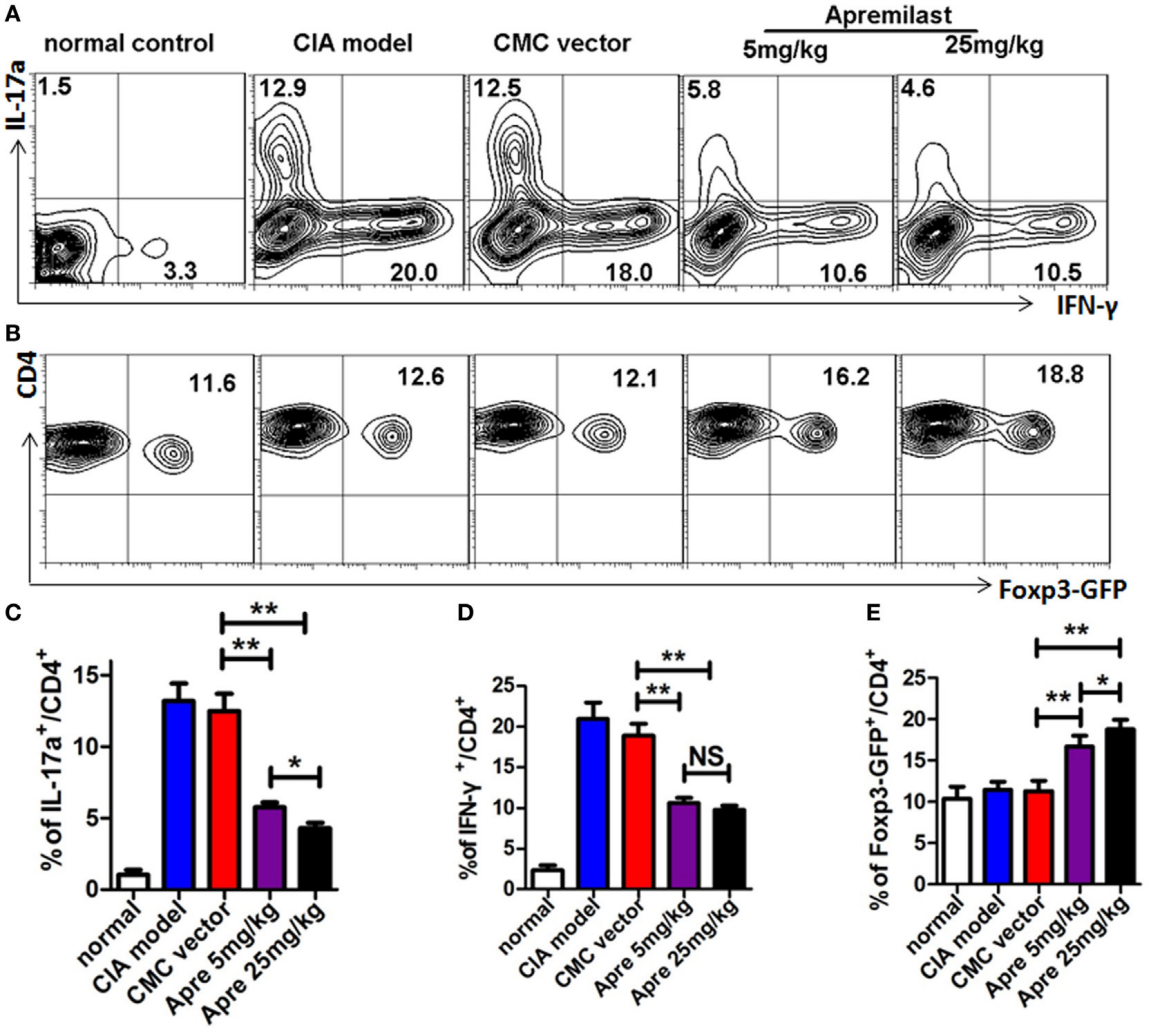
Apremilast reduced arthritis through suppression of Th1 and Th17 cells and enhancement of regulatory T cell differentiation. Cells were isolated from the draining lymph nodes of arthritic mice on day 56 after type II collagen immunization in Apremilast (5 or 25 mg/kg), CMC vehicle alone, collagen-induced arthritis (CIA) model, and normal groups. The expression of IL-17a, IFN-γ **(A)**, and Foxp3^+^-GFP **(B)** on CD4^+^ T cells were measured in draining lymph node cells by flow cytometry. The summary data are shown in the lower panel **(C–E)**. The data indicate the mean ± SEM of five mice per group from two independent experiments (*p < 0.05, **p < 0.01). Data were analyzed using the one-way ANOVA for comparison among multiple groups, followed by Turkey’s test.

As the Th1 and Th17 cytokines are the most important pro-inflammatory response involved in the development of CIA, we next investigated the impact of Apremilast on these effector cells. The analysis clearly demonstrated that Th17 cells and Th1 cells from draining lymph nodes were both decreased in the low and high dose Apremilast groups (Figures 4A,C,D) (Figure S2 in Supplementary Material). No difference between two groups was observed. Foxp3^+^ Treg cells play a crucial role in the maintenance of immune tolerance and prevention of RA. We therefore tested whether Foxp3^+^ Treg cells might also by affected by the Apremilast treatment in CIA. We noted that Treg cells were enhanced in both the low and high dose Apremilast groups (Figures 4B,E). High dose Apremilast treatment resulted in a higher enhancement of Treg cells in CIA (Figures 4B,E) (Figure S2 in Supplementary Material).

### Apremilast Promoted Treg by Maintaining the Foxp3 Stability and Prevented Treg Conversion to Th17 Cells *In Vitro*

**Figure 5:**
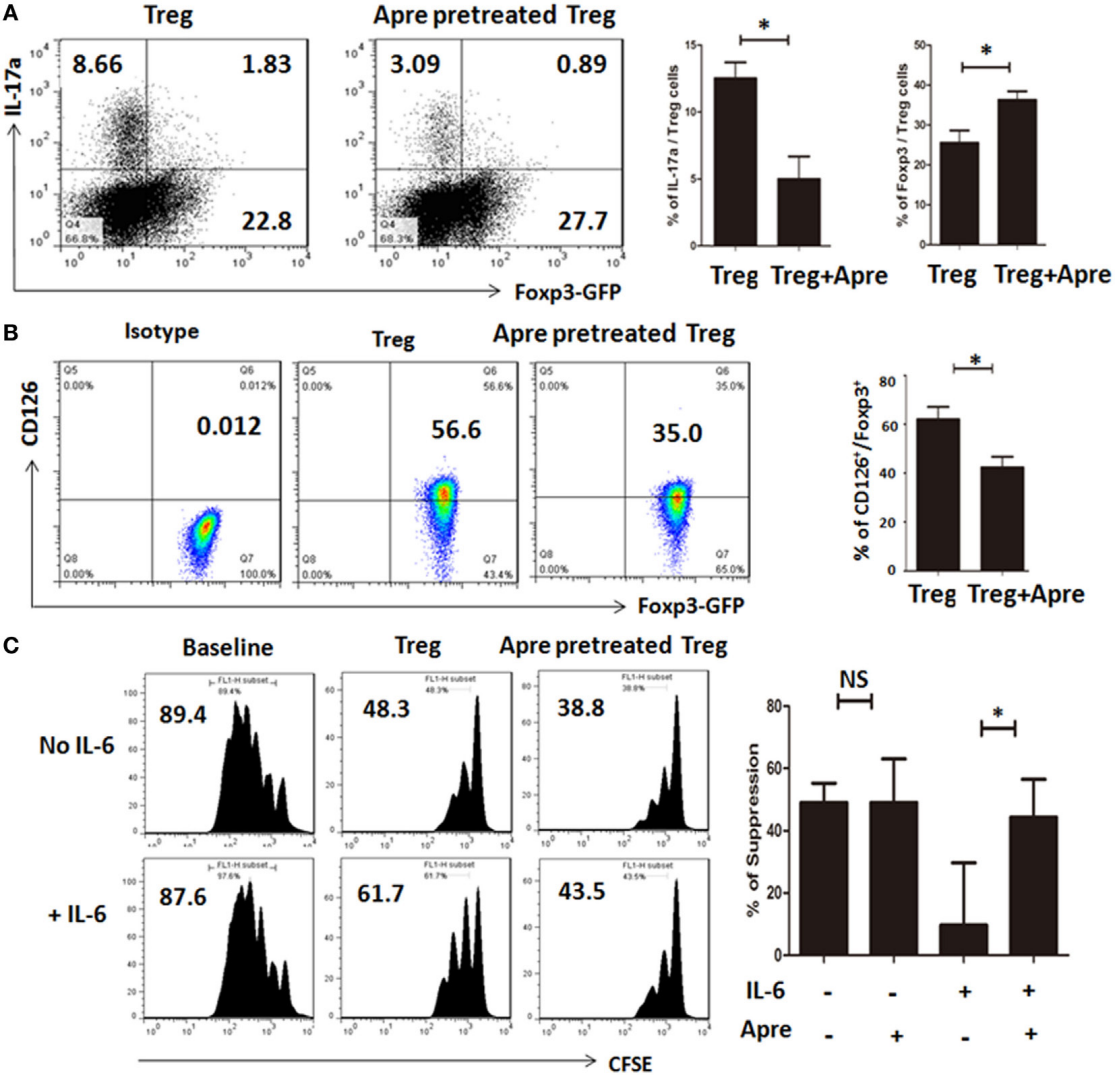
Apremilast promoted Treg by maintaining the Foxp3 stability and prevented Treg conversion to Th17 cells *in vitro*. **(A)** CD4^+^CD25^+^ cells (Treg) were isolated with cell sorting and pretreated with 0.1 µM Apremilast or DMSO for 24 h, cultured under a condition polarizing Th17 cells for another 3 days. Then, the cells were harvested, tested for IL-17a and Foxp3-GFP expression by flow cytometry (mean ± SEM of three independent experiments, Mann–Whitney). **(B)** The CD126 expression was measured on Treg after pretreatment with 0.1 µM Apremilast or DMSO by flow cytometry and qPCR (mean ± SEM of three independent experiments, Mann–Whitney). **(C)** CFSE-labeled T cells were cultured with Treg (pretreated with DMSO) or Treg (pretreated with 0.1 µM Apremilast) in the presence of IL-6 or not at a 1:1 ratio, and suppression of cycling CFSE-labeled T cells was assessed flow cytometry (mean ± SEM of three independent experiments, Mann–Whitney) (*p < 0.05).

We also determined the mechanisms thereby Apremilast promoting Treg cells. We isolated the natural Treg cells from spleen of DBA1 mice, pretreated with Apremilast or DMSO 24 h, then cultured under a condition polarizing Th17 cells for 3 days *in vitro*. We demonstrated that Treg pretreated with Apremilast had a higher Foxp3 expression and lower IL-17a expression when compared with DMSO group under the simulation of IL-6 (Figure 5A). As we known, IL-6 can induce natural Treg to become Th17 cells through IL-6R (CD126) mediated signaling (8, 13, 18). We found that the CD126 expression was quite lower in Treg pretreated with Apremilast than that pretreated with DMSO control (Figure 5B). Furthermore, Treg pretreated with Apremilast or DMSO had a similar function in suppressing the T cell proliferation without IL-6 treatment. However, the ability of Treg to suppress the T cell proliferation was significantly decreased after stimulation with IL-6, while Treg pretreated with Apremilast mostly sustained the suppressive activity in the condition of pro-inflammatory IL-6 (Figure 5C).

### Apremilast Treatment Inhibited RASFs from Migrating and Destroying Cartilage in the Humanized Animal Model

**Figure 6:**
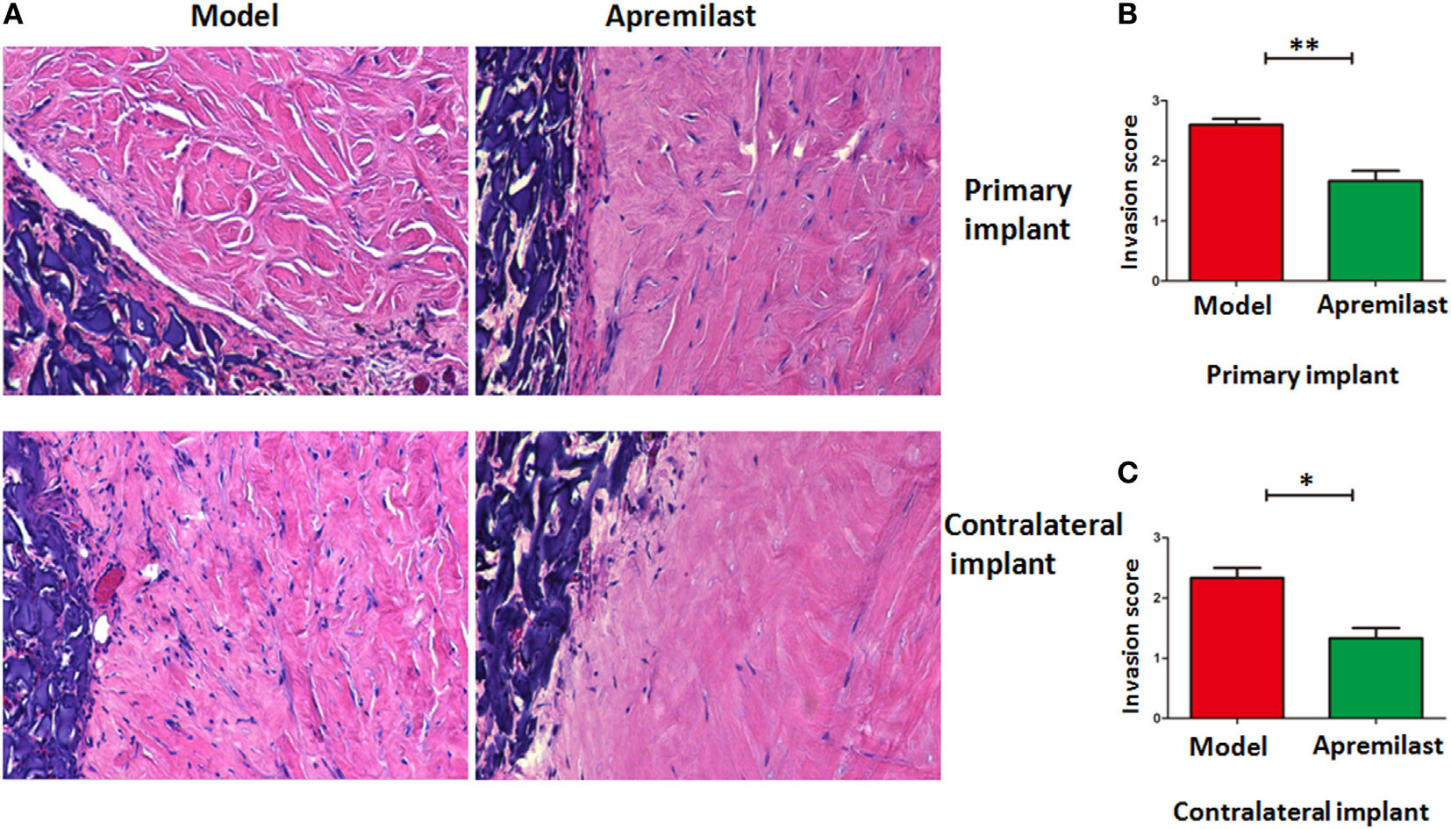
Apremilast treatment inhibited rheumatoid arthritis (RA) synovial fibroblast (RASFs) from migrating and destroying cartilage in the humanized animal model. Sponge-cartilage complex, containing cartilage with synovial fibroblasts from RA patients (RASFs), was implanted into the flank skin of a severe combined immunodeficiency (SCID) mouse (primary implant). We also inserted cartilage without RASFs under the skin of the contralateral flank (contralateral implant). After the humanized synovitis model was established, Apremilast (25 mg/kg, oral, once daily) was given to SCID mice for 10 continuous days. We removed the implants for evaluation after 60 days. Invasion scores are classified as the previous report. **(A)** Apremilast treatment can inhibit the ability of RASFs from migrating to and destroying cartilage. **(B,C)** Compared with the model group (*n* = 6), Apremilast treatment (*n* = 6) significantly reduced the invasion scores in both primary implant and contralateral implant. The data indicate the mean ± SEM from two independent experiments (*p < 0.05, **p < 0.01). Data were analyzed using unpaired *t*-tests (Mann–Whitney).

To determine its clinical relevance, we also developed a humanized animal model to test whether Apremilast can modulate human inflamed synovial tissue-mediated disease *in vivo*. We implanted a sponge-cartilage complex, containing cartilage and RASFs to determine whether Apremilast can suppress the human RASFs function *in vivo*. Cells were phenotyped by flow cytometry to confirm *bona fide* RASFs (Figure S1 in Supplementary Material). After the humanized synovitis model was established, Apremilast (25 mg/kg, once daily) was given to SCID mice for the continuous 10 days. The cartilage damage and scores were evaluated as previously described in Ref. (17). We demonstrated that Apremilast administration significantly suppressed cartilage destruction and RASFs migration (Figure 6), processes that both are relevant to RA.

## Discussion

In this study, our results demonstrated that oral Apremilast therapy delayed arthritis onset and reduced arthritis scores in the CIA model with a dose-dependent effect. Crucial pathogenic antibodies of anti-CII IgG, IgG1, IgG2a, and IgG2b were all reduced after Apremilast administration. Notably, Apremilast markedly prevented bone erosions in CIA mice. These results imply that orally administered Apremilast should be evaluated as a treatment option in patients with RA.

A previous report has shown that intraperitoneal injection of Apremilast can ameliorate arthritis (19). However, this study did not investigate whether Apremilast can prevent bone loss in CIA. Given oral administration is clinically feasible when translating the work to human studies, our study using oral administration adds evidence for the future study of Apremilast treatment in patients with RA.

Given T effector cells (Th1 and Th17) predominately affect the pathogenesis and development of autoimmune arthritis (15, 20), we investigated the hypothesis that Apremilast suppressed Th17 cells and Th1 cells. As expected, Apremilast significantly decreased the frequency of these pathogenic T effector cells. Conversely, Apremilast upregulated the frequencies of Foxp3^+^CD4^+^ Treg cells. The Treg/Teff cell balance is crucial for the development and progression of autoimmune arthritis and other autoimmune diseases (6, 21, 22). Furthermore, we also demonstrated that Apremilast could promote Treg cells by maintaining the Foxp3 stability and preventing Treg cells conversion to Th17 cells *in vitro*. Moreover, Apremilast affects IL-6/IL-6R signaling on Treg cells.

Our study has demonstrated clear effectiveness and a dose–response of Apremilast in the CIA model. While Apremilast has been approved to treat patients with psoriatic arthritis, its therapeutic effect on patients with RA has also been explored in a double-blinded, placebo-controlled phase 2 clinical trial in patients with active RA who were inadequate responders to methotrexate (MTX). Apremilast efficacy was not observed in these patients. The trial did show that Apremilast was well tolerated in patients with longstanding active RA with mean disease duration of greater than 8 years (23). Reasons for this discrepancy between mouse models and patients with RA could be explained with some reasons. Apremilast targets predominantly the Th1 and Th17 response that is important in early RA. However, after the disease is established with a humoral response and the synovial pannus formation, Apremilast may not be as effective. Another possibility for the discrepancy is that the dose used in the clinical trials was too low as a previous study has demonstrated that Apremilast can reduce TNF-α production by human synovial cells from RA patients undergoing joint replacement surgery (19). Additional explanation for the lack of efficacy of Apremilast in RA patients who are MTX inadequate responders is that there is a partial overlap of the mechanisms of action of MTX and Apremilast (24). The PDE4D immunostaining within RA synovia was not elevated in the subgroup of patients with poor response to MTX therapy (25). It is possible that Apremilast may be more appropriate for a different RA population. Further clinical trials are needed to investigate the efficacy of Apremilast in patients with RA with a focus on patients with the early newly diagnosed disease.

Taken together, our study demonstrates that oral administration of Apremilast can ameliorate nonclinical arthritis and can protect against bone damage. We also found that Apremilast regulates the balance between Treg cells and Teff cells in the CIA. These observations may inform the design of clinical trials of Apremilast in patients with early RA, for example prior to MTX exposure, when Th1, Th17, and Treg modulation *via* PDE4 inhibition may have a greater impact.

## Ethics Statement

All animals were treated according to the National Institutes of Health guidelines for the use of experimental animal with the approval of PSU Hershey and the first affiliated hospital at Zhejiang University for the Use and Care of Animals. Patients with RA were recruited with consent from the first affiliated hospital at Zhejiang University with IRB approval.

## Author Contributions

SZ: conception and design of the study; final approval of manuscript; WC, JW, ZX, WQ, JM, FH, HW, GL, and JL: performed experiments; WC, ZX, and SZ: data analysis and interpretation; WC and SZ: wrote the manuscript; RJ and PS: critically revised the manuscript.

## Conflict of Interest Statement

The authors declare that the research was conducted in the absence of any commercial or financial relationships that could be construed as a potential conflict of interest.

## Supplementary Material

Figure S1 Phenotypic characteristic of and rheumatoid arthritis (RA) synovial fibroblasts (RASFs) gained from patients with active RA. RASFs were isolated from digested synovial tissue from two patients with RA. The following fluorescence-conjugated mouse anti-human antibodies were used: PE-anti-CD55 and AlexaFluor 647-anti-CD90. Cell subset was stained with mAbs indicated above and analyzed on a FACS Calibur flow cytometer.

Figure S2 The absolute number of Th17, Th1, and Treg cells in the draining lymph node. The collagen-induced arthritis treated with CMC (vector control, *n* = 5), Apremilast (5 mg/kg, *n* = 5), or Apremilast (25 mg/kg, *n* = 5). The lymphocytes were harvested from the draining lymph node on day 56 after CII immunization. The absolute number was calculated by multiplying whole lymphocytes number by CD4^+^ T cells percentage in whole lymphocytes and the percentage of Th17, Th1, and Treg cells in CD4^+^ T cells. Data were analyzed using the one-way ANOVA for comparison among multiple groups, followed by Turkey’s test (**p* < 0.05, ***p* < 0.01).
