# Supplementary figures and images for: Apremilast Ameliorates Experimental Arthritis via Suppression of Th1 and Th17 Cells and Enhancement of CD4+Foxp3+ Regulatory T Cells Differentiation
Source: Front Immunol. 2018 Jul 18;9:1662. doi: 10.3389/fimmu.2018.01662 (PMC6058600; doi:10.3389/fimmu.2018.01662)

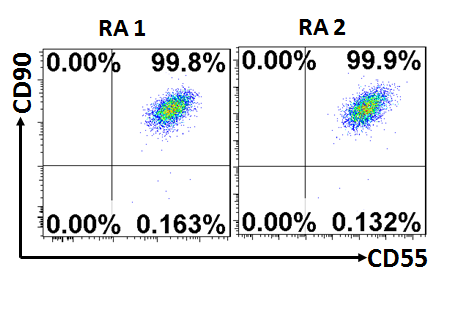

Supplement: Figure S1 — Phenotypic characteristic of and rheumatoid arthritis (RA) synovial fibroblasts (RASFs) gained from patients with active RA. RASFs were isolated from digested synovial tissue from two patients with RA. The following fluorescence-conjugated mouse anti-human antibodies were used: PE-anti-CD55 and AlexaFluor 647-anti-CD90. Cell subset was stained with mAbs indicated above and analyzed on a FACS Calibur flow cytometer. [file Image_1.tif]

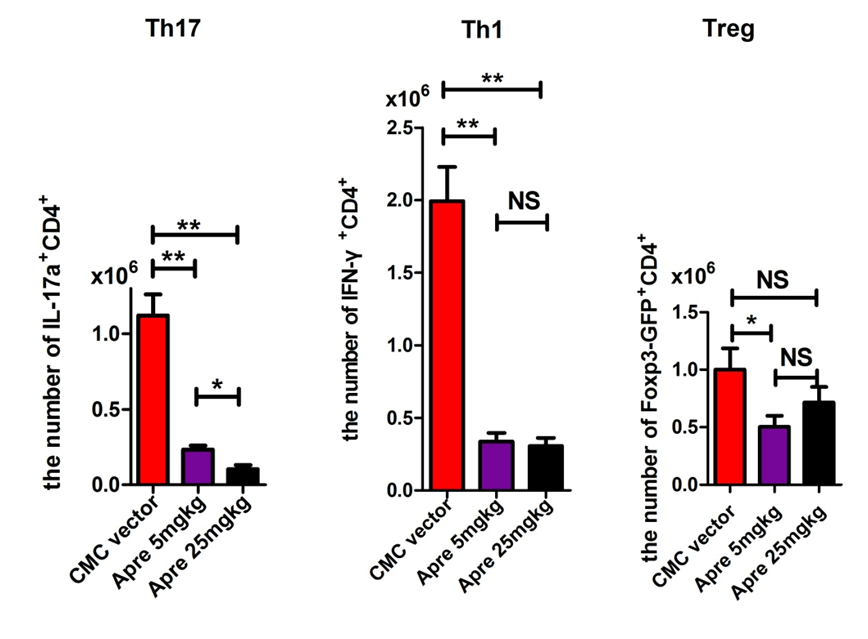

Supplement: Figure S2 — The absolute number of Th17, Th1, and Treg cells in the draining lymph node. The collagen-induced arthritis treated with CMC (vector control, n = 5), Apremilast (5 mg/kg, n = 5), or Apremilast (25 mg/kg, n = 5). The lymphocytes were harvested from the draining lymph node on day 56 after CII immunization. The absolute number was calculated by multiplying whole lymphocytes number by CD4+ T cells percentage in whole lymphocytes and the percentage of Th17, Th1, and Treg cells in CD4+ T cells. Data were analyzed using the one-way ANOVA for comparison among multiple groups, followed by Turkey’s test (*p < 0.05, **p < 0.01). [file Image_2.tif]
